# Supplementary material for: Spatial structure impacts adaptive therapy by shaping intra-tumoral competition
Source: Commun Med (Lond). 2022 Apr 25;2:46. doi: 10.1038/s43856-022-00110-x (PMC9053239; doi:10.1038/s43856-022-00110-x)
Supplement: Supplementary file 2 — Supplementary Material [file 43856_2022_110_MOESM2_ESM.pdf]

Supplementary Information: Spatial Structure Impacts Adaptive Therapy by Shaping Intra-Tumoral Competition

Maximilian A. R. Strobl, Jill Gallaher, Jeffrey West, Mark Robertson-Tessi, Philip K. Maini and Alexander R. A. Anderson

April 15, 2022

Supplementary Methods 1: Stochastic simulation algorithm

A flow-chart of our model is shown in Figure 1b. We implement this model using the following fixed time-step stochastic simulation algorithm:

while $t \leq t_{\text{End}}$ **do**
  for all `cells in the population` **do**
    `Select parameter set (`$k =$ `sensitive or resistant)`
    `Let` $z_1 \sim \mathcal{U}(0,1)$
    if $z_1 \leq (r_k + d_k)\,dt$ **then** ▷ Check if an event occurs
      `Let` $z_2 \sim \mathcal{U}(0,1)$
      if $z_2 \leq \frac{r_k}{r_k+d_k}$ **then** ▷ Check if cell divides
        if cell has space in neighbourhood **then**
          `Let` $z_3 \sim \mathcal{U}(0,1)$
          `Let` $\sigma = 1$ `if the cell is sensitive, otherwise` $\sigma = 0$
          if $z_3 \leq \sigma d_D D$ **then** ▷ Drug-induced death
            `Cell dies from drug`
          else ▷ Cell division
            `Cell divides into randomly chosen spot in neighbourhood`
      else ▷ Check if cell dies
        `Cell dies`
  $t \leftarrow t + dt$

Here, $\mathcal{U}(0,1)$ denotes the uniform distribution on $[0,1]$, and t_{End} the end time of the simulation in days.

Supplementary Methods 2: Consistency analysis

A consistency analysis serves to determine the number of independent replicates required to obtain a sample of outcomes representative of the stochastic process. We adopted the protocol from [1] and applied it to corner cases of our parameter space, (n_0, f_R, c_R, d_T). The idea is to choose a sample size, n, obtain k independent samples of this size, and compare the k distributions of model outcomes. The aim is to find a value of n so that the k distributions are sufficiently similar and any one of them is representative of the others. We computed the difference in TTP for adaptive and continuous therapy for 10 samples of sample sizes $n = 10, 50, 100, 250, 500, 1000$, and 1500, respectively $(68, 200$ simulations total). In Supplementary Figure 1a we illustrate, for one parameter combination, how the thus obtained 10 outcome distributions for each value of n become almost indistinguishable for $n \geq 250$. To quantify consistency we measured the mean value of each distribution and the proportion of runs in which adaptive therapy performed worse than continuous therapy. This corroborates that $n \geq 250$ generates outcome distributions with very similar mean values and lower tails for a range of parameter combinations (Supplementary Figure 1b & c). However, some parameter sets converge more slowly than others. Thus, we choose a value of $n = 1000$ for all analyses except for time-series plots (e.g. Figure 2a & b) for which we choose $n = 250$ for computational reasons.

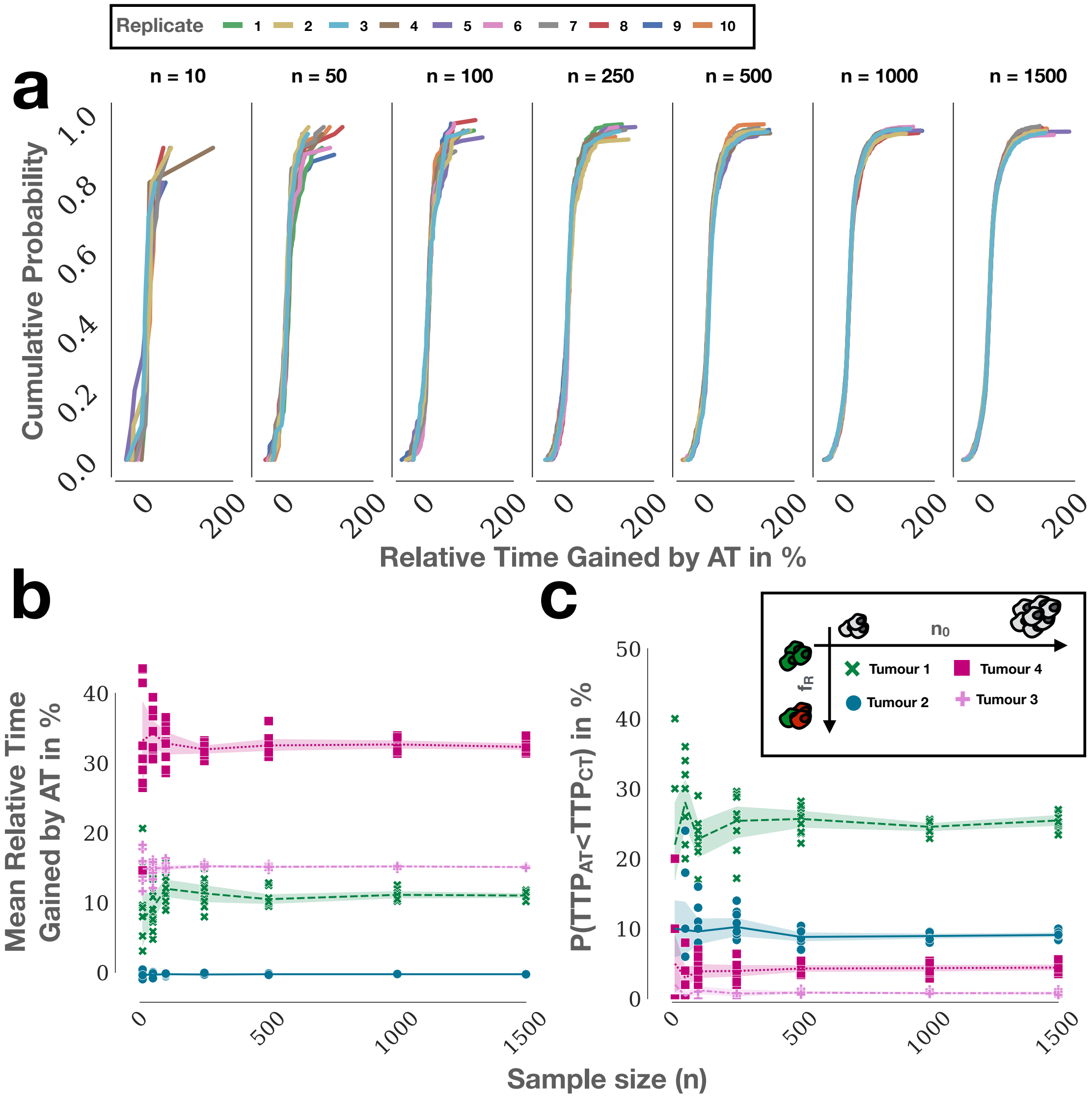

Supplementary Figure 1: Representative results of the consistency analysis: (**a**) Increasing consistency in the distribution of time gained by adaptive therapy for one parameter set $((n_0, f_R, c_R, d_T) = (25\%, 0.1\%, 0\%, 30\%))$ as the number of independent replicates per sample, n, is increased. For each value of n, $k = 10$ independent samples of sample size n were collected. (**b**) Mean value of the outcome distribution as a function of sample size, n, for four parameter sets (Tumour 1: $(n_0, f_R) = (25\%, 0.1\%)$; Tumour 2: $(n_0, f_R) = (25\%, 10\%)$; Tumour 3: $(n_0, f_R) = (75\%, 10\%)$; Tumour 4: $(n_0, f_R) = (75\%, 0.1\%)$). Lines indicate the mean value, shading a 95% confidence interval. Markers show values of individual replicates. For sample sizes upwards of 250, the different replicates produce very consistent results. (**c**) Proportion of runs in which adaptive therapy failed as a function of the sample size, n, for the same four parameter sets. Again for $n \geq 250$ we see consistent values between independent replicates indicating that a sample size $n \geq 250$ will yield a representative outcome distribution.

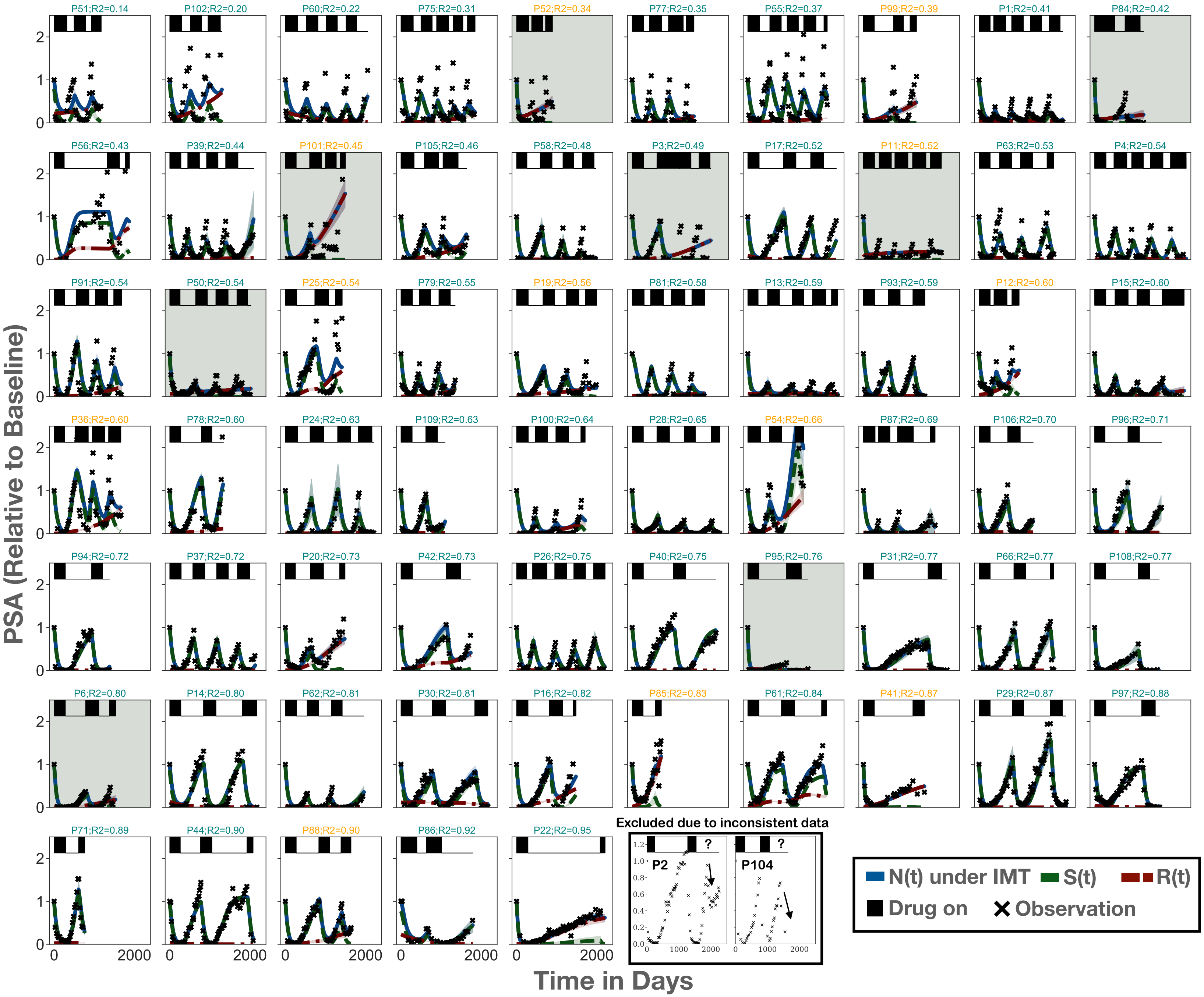

Supplementary Figure 2: Overview of the ABM fits of the full model (all 4 parameters) for all 65 patients, arranged by their r^2 value (showing the mean and standard deviation of 25 replicates per patient). Title colour indicates whether a patient relapses (orange) or not (green). Patients who were excluded due to poor model fits from the correlation analysis between model parameters and cycling speed are marked with a grey background. The inset in the bottom right corner shows the two patients who were excluded from the analysis because their PSA dynamics were inconsistent with the reported treatment schedules.

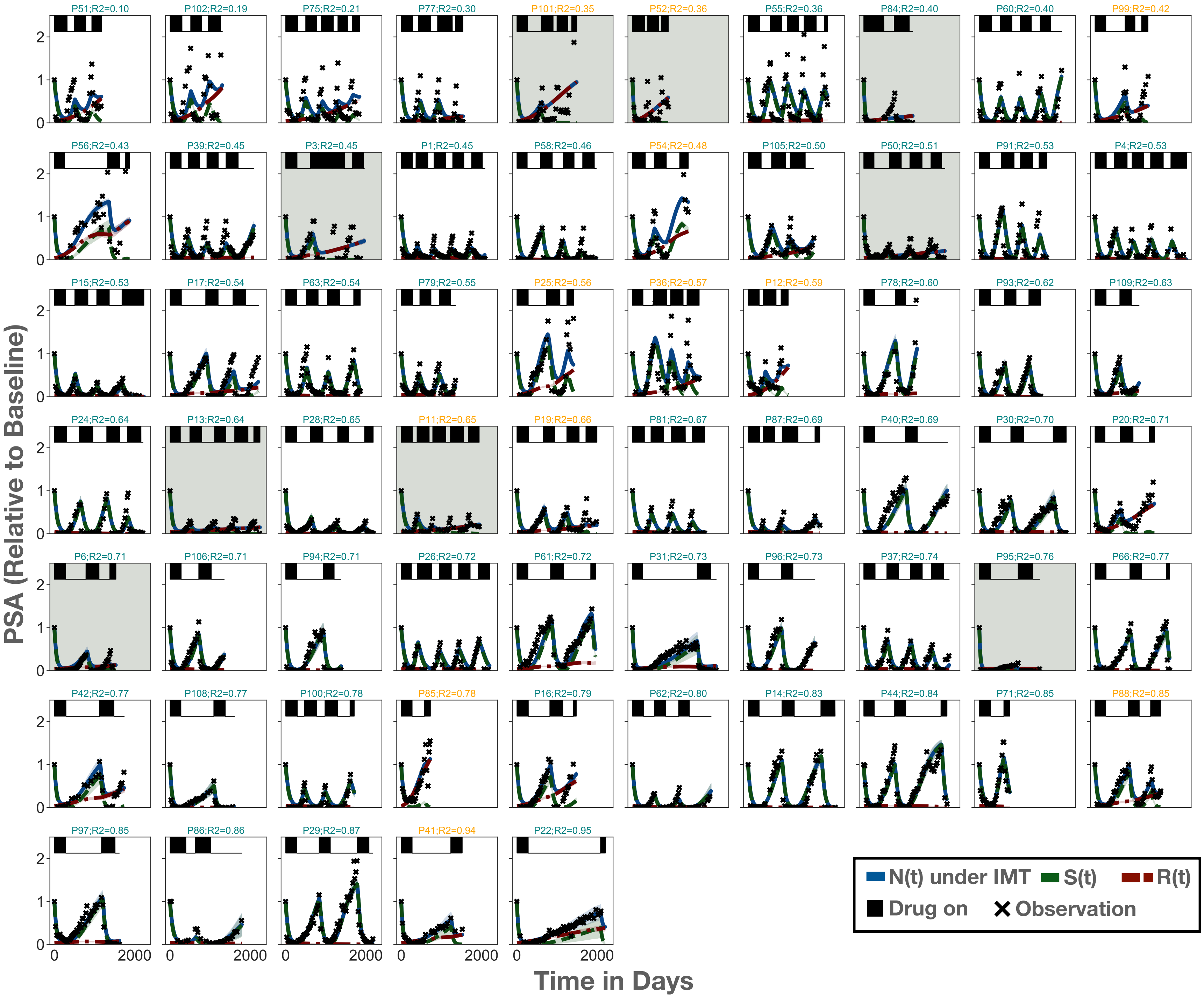

Supplementary Figure 3: Overview of the ABM fits of the reduced model (fitting only cost and turnover) for all 65 patients, arranged by their r^2 value (showing the mean and standard deviation of 25 replicates per patient). Title colour indicates whether a patient relapses (orange) or not (green). Patients who were excluded due to poor model fits from the correlation analysis between model parameters and cycling speed are marked with a grey background.

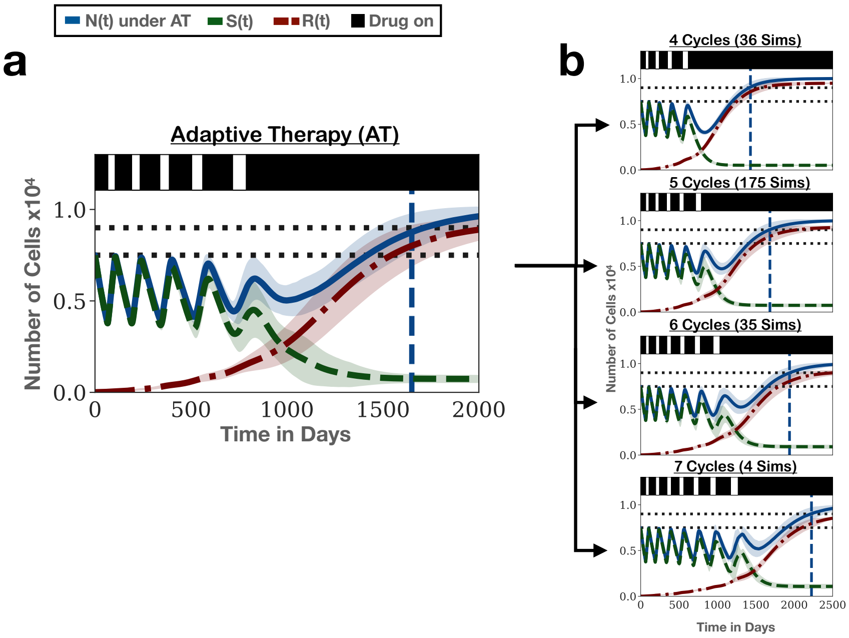

Supplementary Figure 4: Example of the variation in the adaptive therapy treatment dynamics due to the stochastic nature of the simulations ((n_0, f_R, c_R, d_T)=(75%,0.1%,0%,0%)). (**a**) Adaptive therapy treatment dynamics across all 250 independent simulations, as displayed in Figure 2a. Shown are the mean and standard deviation (shading) of the tumour cell numbers. Black bars here and throughout the paper illustrate the treatment schedule with the most common number of cycles. Horizontal dotted lines show the initial cell number, and the cell number at progression. Vertical lines and associated shading mark the mean, and the 1st and 3rd quartile of the distribution of TTP. (**b**) Simulations grouped by the total number of adaptive therapy cycles undergone. Despite having identical model parameters there is noticeable variability in the response to AT between replicates, due to differences in the initial seeding of cells and the stochastic nature of the simulations. This indicates that stochastic effects may also play a role in determining the outcome of adaptive therapy, although owing to the unrealistically small number of cells in our simulations our results allow no quantitative conclusion about the magnitude of this effect.

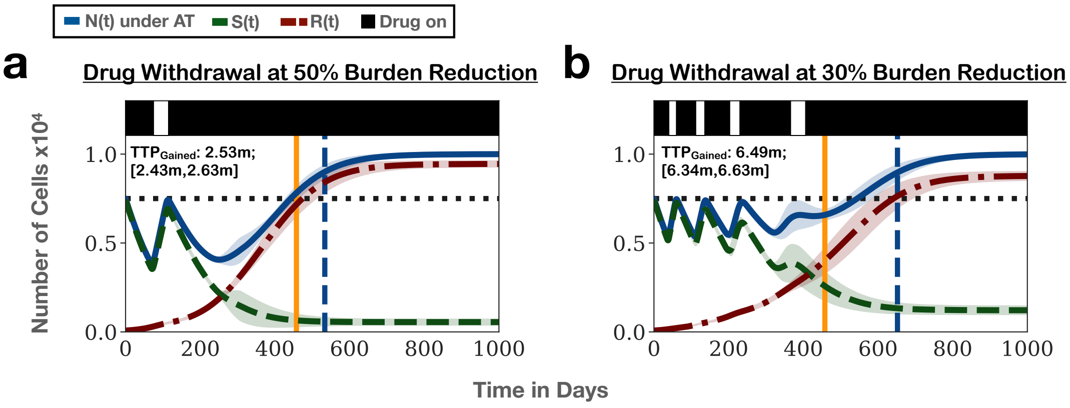

Supplementary Figure 5: Comparison of two adaptive therapy protocols with different thresholds for treatment withdrawal. (**a**) Treatment withdrawal after a 50% tumour burden reduction. Lines and shading show the mean and standard deviation of the number of cells in each subpopulation and the total tumour size, respectively. Vertical lines denote the time of progression under continuous (yellow) and adaptive therapy (blue). Inset gives mean time gained and 95% confidence interval. (**b**) Treatment withdrawal after a 30% tumour burden reduction. This suggests that less aggressive treatment, especially if resistance is prevalent prior to treatment, will result in longer tumour control, and matches the results obtained for non-spatial models [2, 3, 4, 5]. Note though that we are not taking into account possible risks associated with the higher tumour burden under the 30% threshold algorithm. Parameters: $(n_0, f_R, c_R, d_T) = (75\%, 1\%, 0\%, 0\%)$; $n = 250$ independent replicates.

Supplementary Discussion 1: Comparison of random initial seeding with seeding as a circular domain

Throughout the main paper we initialise our simulations by seeding cells randomly in the domain. This was meant to recapitulate the heterogeneous structure of tumours in which nests of tumour cells are interspersed with remnants of normal tissue, tumour stroma, or areas of necrosis. In addition, it means that resistant cells are not guaranteed to be surrounded by sensitive cells, so that this appeared to be a good "worst case" scenario in which to test adaptive therapy. However, a more common way in which tumours are modelled in the literature is as discs (in 2-D) or balls of cells (in 3-D), in which the tumour grows radially outwards from its site of origin and the majority of cell division takes place on the surface of the growing tumour (e.g. [6, 7]).

To examine how our conclusions are affected by such spherical tumour architecture, we repeated some of our analyses with the cells seeded as a disc in the centre of the domain, surrounded by empty space. To allow comparison with the random initial seeding in the main paper, we chose the radius of this disk to be given by:

$$r = \texttt{floor}\left(\sqrt{\frac{n_0 10^4}{\pi}}\right),$$

so that approximately the same total number of cells was seeded. We then seeded exactly the same number of resistant cells as in the random case within this disk ($R_0 = f_R n_0 10^4$), and filled up the rest of the disk with sensitive cells. Furthermore, in order to allow cells on the surface to expand freely, we increased the domain size to $l = 150$px.

Supplementary Figure 6a shows a comparison of the relative time gained by adaptive therapy over continuous therapy for the two types of initial conditions. We find that which of the two is predicted to benefit more from adaptive therapy depends on the initial cell density (n_0), the initial resistant cell fraction (f_R), and the cell turnover (d_T) in a multi-factorial fashion. Supplementary Figure 6b shows the treatment response when we assume the same parameters as in Figures 2a-c, but seed the cells in a circular fashion. In this case, the benefit of adaptive therapy is greatly diminished, as resistant cells near the surface of the tumour are freed early during treatment and can subsequently expand unobstructed towards the outside (Supplementary Figure 6b; centre panel).

However, while seeding the cells in this way means that clones on the outside can expand more freely, it also means that cells are in stronger competition with each other at the beginning of the simulation (compare the right panel in Supplementary Figure 6b with Figure 2c). As a result, when we start with a small number of cells (small n_0) adaptive therapy is more effective when cells are seeded in a circular fashion than when they are placed randomly (Supplementary Figures 6a & c).

A further implication of this increased competition is that turnover has a greater beneficial effect on adaptive therapy when cells are seeded as a disk than when they are seeded randomly (Supplementary Figures 6a & d). This is because turnover means that drug can kill cells throughout the tumour, so that not as much of the cell mass near the surface needs to be removed to achieve the 50% burden reduction required for treatment withdrawal (compare tumour sizes at $t = 500$d in centre panels in Supplementary Figures 6b & d). Consequently, resistant nests near the edge remain surrounded by sensitive cells for longer and can, thus, be controlled better than in the random seeding (or no turnover) case.

To sum up, these results corroborate our findings in the paper that the tumour's spatial organisation determines whether, and for how long, resistance may be controlled by adaptive therapy. This is because the architecture shapes the nature of intra-tumoral competition. We advocate that future research should study the impact of different tissue structures more explicitly, using for example the different "onco-evotypes" recently defined by Noble et al [8]. Importantly, such research should also interrogate the role of non-tumour tissue which we have neglected here. First steps in this direction have recently been presented by M A et al [9].

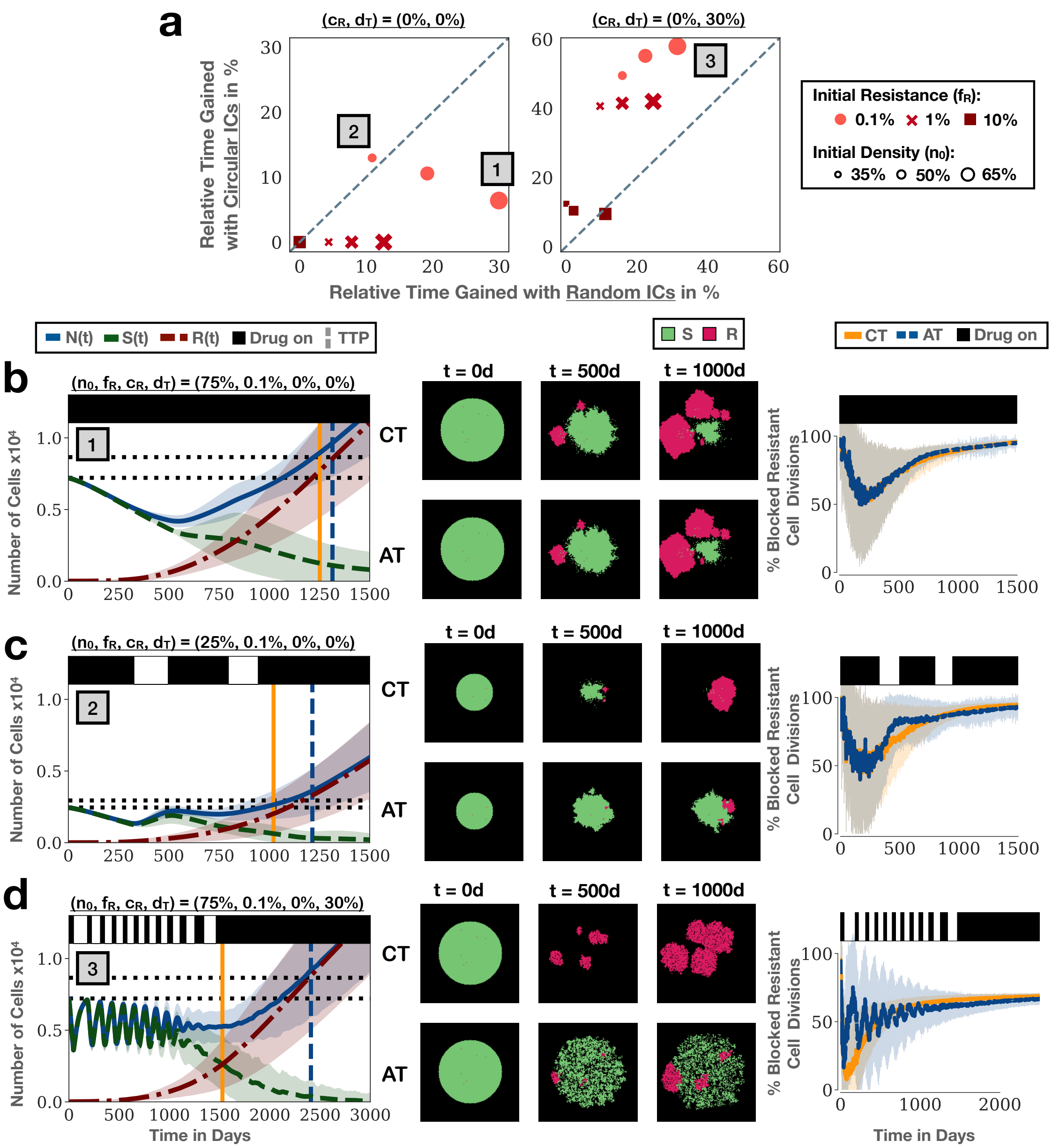

Supplementary Figure 6: The performance of adaptive therapy when tumour cells are seeded as disks of cells in the centre of the domain rather than randomly. (**a**) Relative time gained by adaptive therapy under the two initial conditions (see Supplementary Discussion 1 for details on how simulations were matched). Depending on the initial cell numbers and turnover either random or circular seeding will result in a greater benefit of adaptive therapy. Points show mean values from $n = 1000$ replicates. Grey labels correspond to (b) - (d). (**b**) Treatment trajectory assuming the same parameters as in Figure 2a-c, but with cells seeded as a disk rather than randomly. Adaptive therapy is unable to control resistance as treatment frees resistant colonies near the edge which can subsequently expand in an unhindered way. (**c**) Simulations with the same parameters as (b) but with a smaller initial cell number. The smaller cell number means that the tumour radius has to be reduced by less to hit the 50% threshold at which treatment is withdrawn so that it takes longer for resistant cells to be freed (compare tumour size change between $t = 0$d and $t = 500$d in the middle panels in (b) and (c)). (**d**) Simulations with the same parameters as (b) but with a 30% turnover rate. Turnover means that drug kill is spread more evenly throughout the tumour so that resistant cells near the edge are contained for longer than in the absence of turnover. This corroborates our main finding that the tumour architecture impacts adaptive therapy by changing the competition dynamics of both resistant and sensitive cells. Panels (b) - (d) are based on $n = 250$ independent replicates.

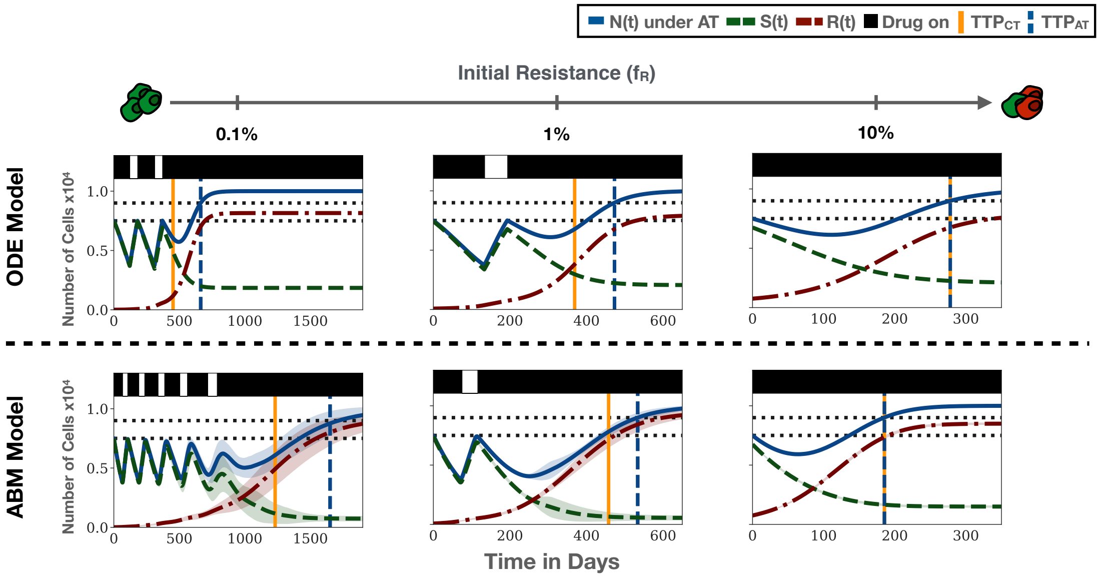

Supplementary Figure 7: Comparison of the treatment predictions of the ABM and ODE model (Equations (2)-(4)) for the same initial density ($n_0 = 75\%$) but different initial resistance fractions. We assume no cost or turnover. For the ABM the mean and standard deviation of $n = 250$ independent replicates are shown. When the initial resistance fraction is small, the ABM predicts significantly longer TTP than the ODE model. When the resistance fraction is large, the converse is true. This indicates that because of the impact of space, different initial numbers of resistant cells (and thus independent colonies) result in distinct progression dynamics in the ABM (see also Figure 5).

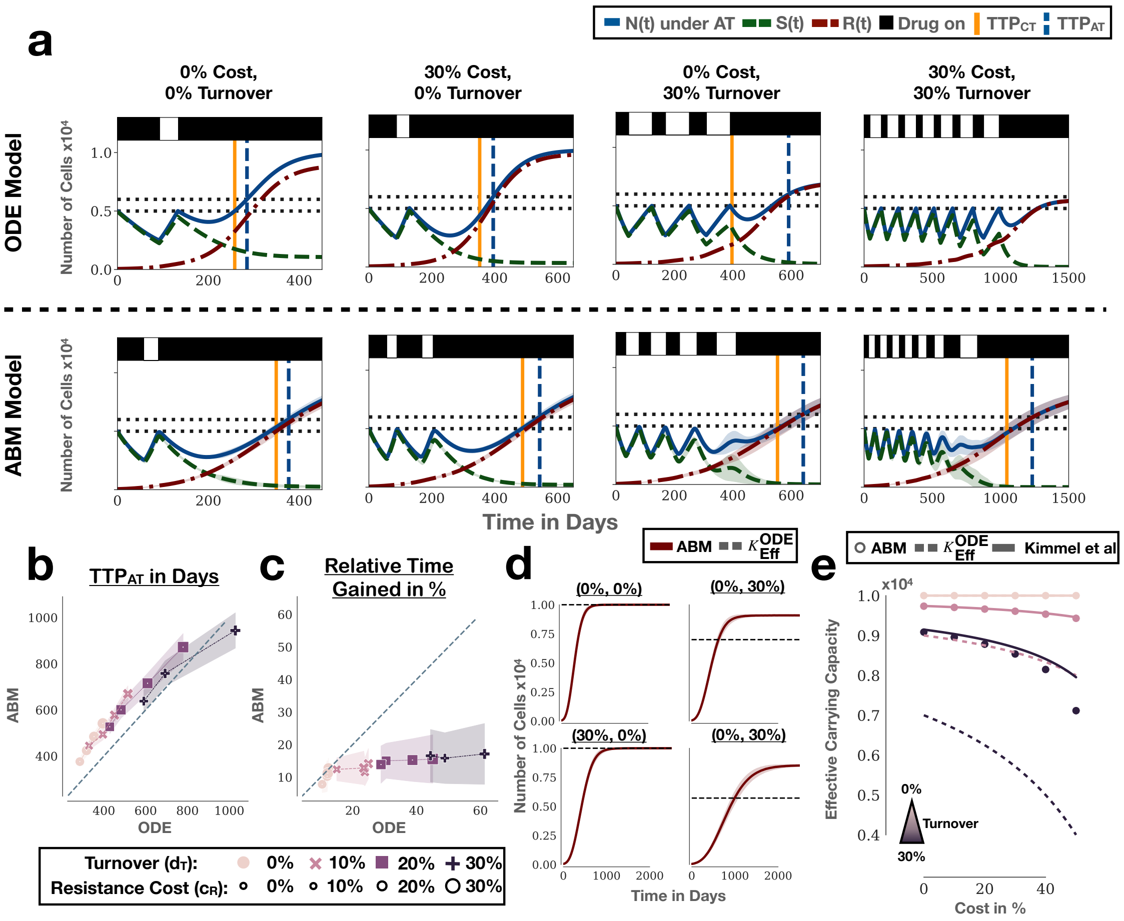

Supplementary Figure 8: Comparison of the treatment predictions of the ABM and ODE model (Equations (2)-(4)) for different values of cost and turnover. Lines denote mean and shading the standard deviation. (**a**) Matched simulations for different combinations of cost and turnover values ($(n_0, f_R) = (50\%, 1\%)$). For the ABM, the mean and standard deviation of $n = 250$ independent replicates are shown. (**b**) Comparison of the TTP under adaptive therapy in the ODE and ABM. The ABM predicts, in general, later progression but this difference becomes smaller in the presence of cost and turnover ($n = 1000$ replicates of the ABM). (**c**) Comparison of the relative time gained by adaptive therapy ($(\text{TTP}_{\text{AT}}\text{-}\text{TTP}_{\text{CT}})/\text{TTP}_{\text{CT}}$) predicted by the two models. The ABM predicts a significantly smaller benefit for adaptive therapy from cost and turnover than the ODE model. (**d**) The reason for the discrepancy observed in (c) is that the impact of turnover on population growth is smaller in the ABM than in the ODE. This can be seen by the fact that the effective carrying capacities, K_{Eff}, (the steady states of the resistant population) are different between the two models in the presence of turnover. Shown are simulations of resistant cells grown in isolation (no drug) under different conditions ($n = 250$ replicates). The dashed line gives the steady state expected in the ODE model ($K_{\text{Eff}}^{\text{ODE}} = \left(1 - \frac{d_T}{r_R}\right) l^2$). We previously showed that K_{Eff} is an important factor in determining the benefit of adaptive therapy [5]. As such, the differences in K_{Eff} explain the discrepancy in the predicted benefit of adaptive therapy. (**e**) The reason why the effective carrying capacities differ is the fact that in the ABM a cell has four potential neighbouring sites into which it can divide. This allows for more division to take place than predicted by the ODE model. To show this, we compare the effective carrying capacity in the ABM for different values of cost and turnover to an expression derived by Kimmel et al [10] which accounts for the neighbourhood size: $K_{\text{Eff}}^{\text{ABM}} \approx \left(1 - \frac{d_T}{r_R}\right)^{\frac{1}{a}} l^2$, where a is the neighbourhood size ($a = 4$ for a von Neumann neighbourhood). With the neighbourhood size taken into account we see excellent agreement with the observed effective carrying capacity in the ABM. Values for the ABM were obtained by taking the final population size after 10y, as in (d) ($n = 1000$ replicates per condition).

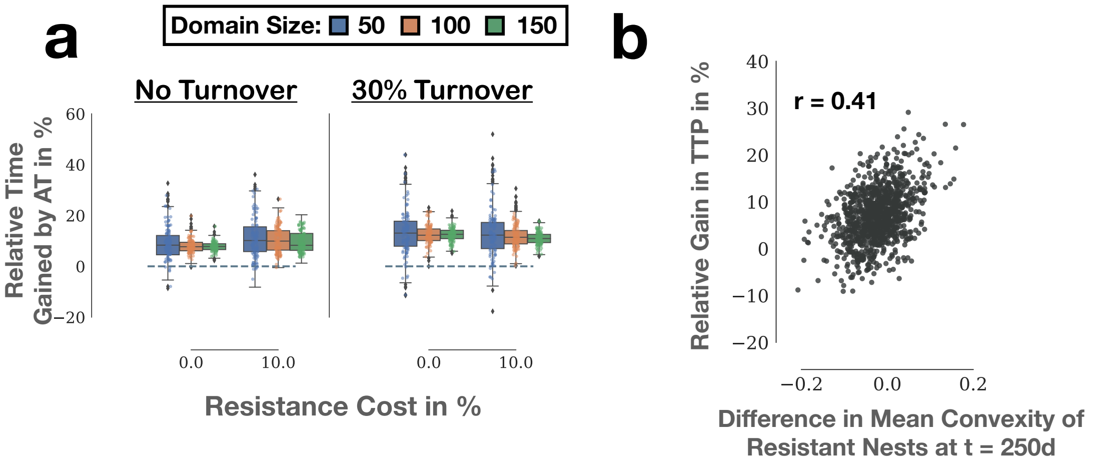

Supplementary Figure 9: Supplemental analyses of why continuous therapy sometimes controls the tumour for longer than adaptive therapy. (**a**) Domain size (l) of the ABM reduces the variance in outcomes, corroborating the importance of the initial cell number and thus stochastic effects ($(n_0, f_R) = (50\%, 1\%)$; $n = 1000$ independent replicates). The box, centre line, and whiskers denote the inter-quartile range, median, and 1.5 x inter-quartile range, respectively. (**b**) In addition, the morphology of the resistant cell nests appears to play a role. Shown is the relative gain of adaptive therapy as a function of the difference between the two treatment arms in the convexity of the resistant cell nests (defined as Convexity $= \frac{\text{Perimeter of Convex Hull}}{\text{Perimeter}}$ [11]; negative differences indicate that continuously treated colonies are rounder than adaptively treated ones) at 250d ($(n_0, f_R, c_R, d_T) = (25\%, 0.1\%, 0\%, 0\%)$; $n = 1000$ independent replicates.). The observed correlation (Pearson's correlation coefficient, $r = 0.41$, p-value < 0.01) indicates that instances in which nests under adaptive therapy are more branched than nests under continuous therapy (smaller convexity values) are associated with poorer performance of AT. To compute a nest's convexity we used first the `connectedComponentsWithStats()` function in `openCV` [12] to identify individual nests, followed by the `convexHull()` function to find their convex hull.

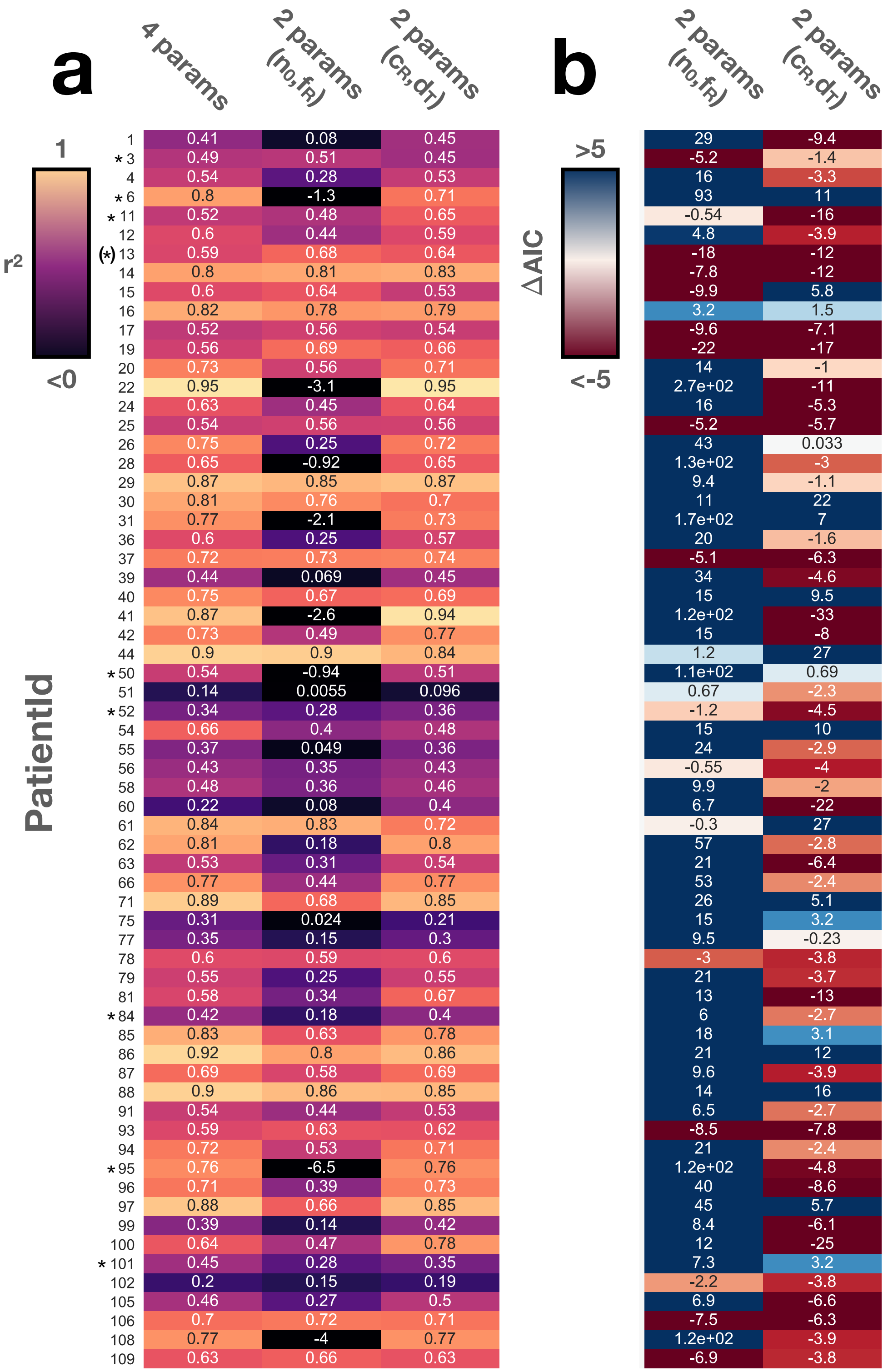

***Patient excluded when computing the global, fixed parameter values when fitting the 2-parameter models, and excluded from the analysis of the correlation between model parameter and cycling speed, because the model fit does not recapitulate the patient's cycling dynamics. Patient 13 was only excluded from the analysis of the 2-parameter model.**

Supplementary Figure 10: Comparison of fitting the ABM by allowing either the initial tumour composition (n_0, f_R), the cell kinetics (c_R,d_T), or all four parameters to vary ("4 params"). (**a**) Comparison of the r^2 values for each patient for each model. We observe that allowing only cost and turnover to be patient-specific can explain the data almost as well as the full 4-parameter model. This is not true for the model in which the initial conditions are patient-specific. Note that the negative r^2 values occur due to the fact that the ABM provides very poor fits in these cases. (**b**) Difference in AIC between the 2-parameter models compared to the 4-parameter models for each patient. The AIC represents a measure of goodness-of-fit relative to a model's complexity. When the AIC of two models differs by more than 2, the model with the smaller AIC is the preferred one [13]. This corroborates that for most patients the cost-turnover model provides the best description of the data.

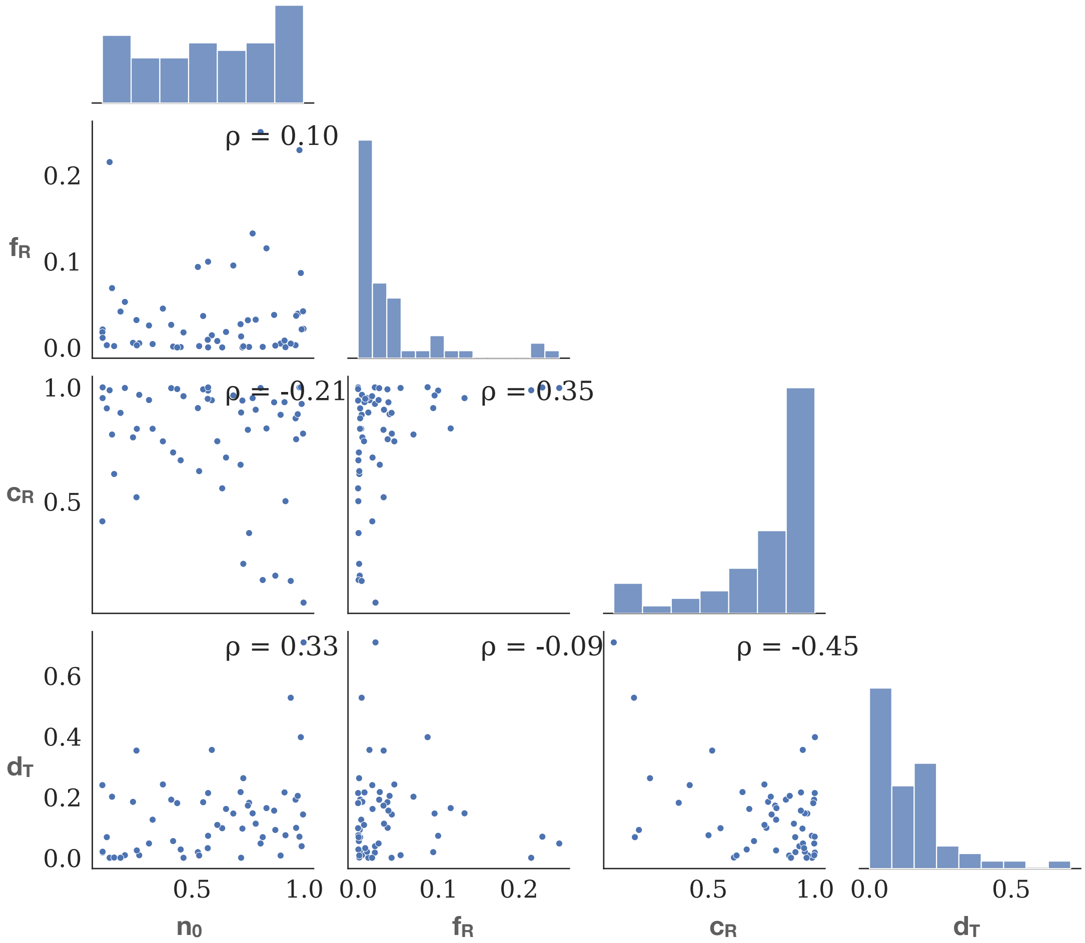

Supplementary Figure 11: Pair-wise correlation between the inferred parameters when fitting the ABM (4-parameter model, allowing n_0, f_R, c_R, and d_T to vary on a patient-specific basis). Each point represents a patient ($n = 57$; the 8 patients for whom the model did not capture the cycling were excluded). Inset text shows the Pearson's correlation coefficient. Histograms on the diagonal illustrate the distribution of each parameter.

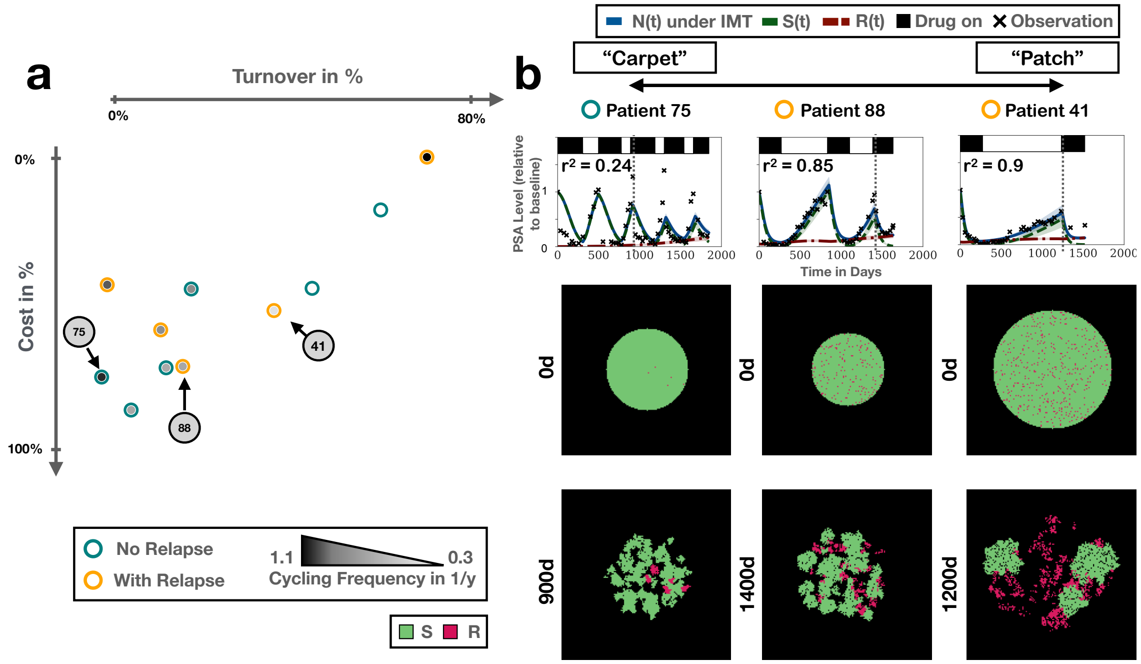

Supplementary Figure 12: Fitting the patient data assuming a disk-like tumour growth model, where the tumour begins as a disk in the centre of the domain and expands radially outwards. Tumours were initialised as described in Supplementary Discussion 1 (except that resistant cell numbers were not matched, so that f_R here represents the true initial resistant fraction) and fitted using the same methods as described in Section 2.4 of the main text, allowing n_0, f_R, c_R, and d_T to vary on a patient-specific basis. Shown are the results for the subset of 11 patients whose dynamics are displayed in Supplementary Video 4. (**a**) Plot of the inferred cost (c_R) and turnover (d_T) values, revealing a correlation between the two values also in this case (Pearson's correlation coefficient: $r = -0.87$, $p < 0.01$). (**b**) Examples of treatment dynamics predicted by the model for three patients ($n = 250$ independent replicates in each case). This shows that, as in the case of random initial seeding, faster cycling patients are associated with more diffuse ("carpet-like") intra-tumoral architecture, whereas patients who are cycling slowly are fitted by simulations in which the dynamics is dominated by few, large sensitive colonies ("patch-like").

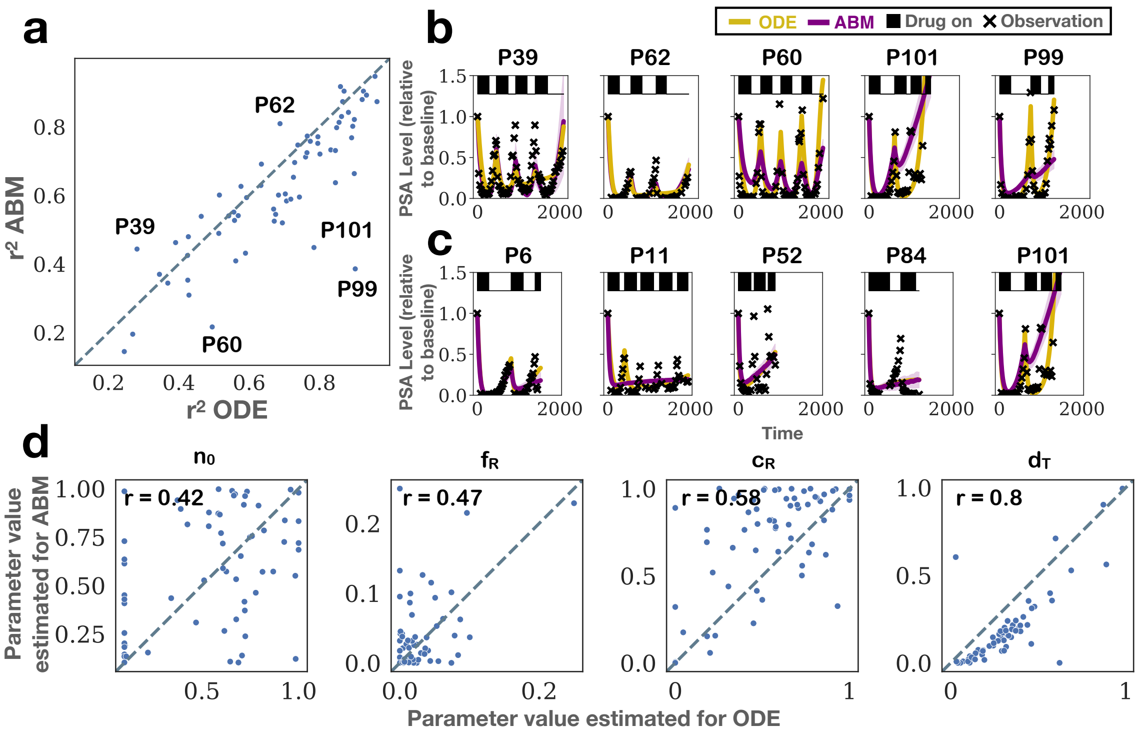

Supplementary Figure 13: Comparison of the patient fits for the ODE and the ABM model (varying n_0, f_R, c_R, d_T). ODE fits taken from our prior work [5]. ABM simulations show mean and standard deviation from $n = 25$ independent replicates. (a) Comparison of the r^2 values of both models, showing that the goodness-of-fit of both models is generally comparable, although the ODE achieves slightly better fits on average (each point is one patient; $n = 65$). This is not unexpected given the extra complexities involved in fitting the ABM, such as stochasticity, longer run times, and gradient-based vs heuristic optimisation. Nevertheless, we believe that our new ABM results provide valuable insights, as the ODE model allowed us to formulate hypotheses about why the patients cycle at different rates only in terms of cell kinetic parameters (e.g. cost and turnover), which are difficult to measure in practice. In contrast, the ABM revealed that differences may additionally manifest themselves in the spatial architectures of the tumours, a hypothesis that is more easily testable, for example, by analysing histology. (b) Comparison of the treatment trajectory predicted by both models for two patients for whom the ABM fits better (P39 and P62), and three patients for whom the ODE achieved better fits (P60, P101, and P99). We can see that the ODE is better able to capture the peak PSA values in some fast cycling patients. (c) ODE and ABM fits for a representative subset of the patients that were excluded from the cycling analysis, because the ABM was unable to capture the cycling dynamics. In all cases the ODE fails to recapitulate the dynamics of these patients, too and, in fact, these patients were also excluded from the analysis in our prior paper [5]. This suggests that the observed dynamics in these patients is not explicable by our simple 2-population model, and that other factors, such as a third tumour cell population or non-tumour cells (immune cells or stroma) may be at play. (d) Comparison of the parameter values inferred by the two models (each point is one patient; $n = 65$). Inset text shows the Pearson's correlation coefficient. There is generally little correlation, likely because of differences in how these parameters act on the growth dynamics in the two models. The exception to this is turnover, indicating an important role in both models. Overall, the ABM results presented here corroborate and complement our prior ODE analysis by providing an interpretation of how the inferred differences in parameter values may manifest in the spatial architecture of the tumours.

References

[1] S. Hamis, S. Stratiev, and G. G. Powathil, “Uncertainty and Sensitivity Analyses Methods for Agent-Based Mathematical Models: An Introductory Review,” in *Physics Of Cancer, The: Research Advances*, World Scientific, 2020.

[2] E. Hansen and A. F. Read, “Modifying adaptive therapy to enhance competitive suppression,” *Cancers*, vol. 12, no. 12, pp. 1–13, 2020.

[3] H. C. Monro and E. A. Gaffney, “Modelling chemotherapy resistance in palliation and failed cure,” *Journal of Theoretical Biology*, vol. 257, no. 2, pp. 292–302, 2009.

[4] Y. Viossat and R. Noble, “A theoretical analysis of tumour containment,” *Nature Ecology and Evolution*, pp. 1–10, 2021.

[5] M. A. Strobl, J. West, Y. Viossat, M. Damaghi, M. Robertson-Tessi, J. S. Brown, R. A. Gatenby, P. K. Maini, and A. R. Anderson, “Turnover modulates the need for a cost of resistance in adaptive therapy,” *Cancer Research*, vol. 81, pp. 1135–1147, 2021.

[6] K. Bacevic, R. Noble, A. Soffar, O. W. Ammar, B. Boszonyik, S. Prieto, C. Vincent, M. E. Hochberg, L. Krasinska, and D. Fisher, “Spatial competition constrains resistance to targeted cancer therapy,” *Nature Communications*, vol. 8, no. 1, pp. 1–15, 2017.

[7] J. A. Gallaher, P. M. Enriquez-Navas, K. A. Luddy, R. A. Gatenby, and A. R. Anderson, “Spatial heterogeneity and evolutionary dynamics modulate time to recurrence in continuous and adaptive cancer therapies,” *Cancer Research*, vol. 78, no. 8, pp. 2127–2139, 2018.

[8] R. Noble, D. Burri, J. N. Kather, and N. Beerenwinkel, “Spatial structure governs the mode of tumour evolution,” *bioRxiv*, 10.1101/586735, doi: https://doi.org/10.1101/586735, 2019.

[9] M. M A, J.-Y. Kim, C.-H. Pan, and E. Kim, “The impact of the spatial heterogeneity of resistant cells and fibroblasts on treatment response,” *PLOS Computational Biology*, vol. 18, no. 3, pp. 1–33, 2022.

[10] G. J. Kimmel, J. West, M. Damaghi, A. R. A. Anderson, and P. M. Altrock, “Local contact inhibition leads to universal principles of cell population growth,” *arXiv*, 2108.10000, doi: http://arxiv.org/abs/2108.10000, 2021.

[11] A. Jain, *Fundamentals of Digital Image Processing.* Englewood Cliffs, NJ: Prentice-Hall, 1986.

[12] G. Bradski, “The OpenCV Library,” *Dr. Dobb’s Journal of Software Tools*, 2000.

[13] W. J. Krzanowski, *An Introduction to Statistical Modelling.* Wiley, 2010.
