# Supplementary material for: Spatial structure impacts adaptive therapy by shaping intra-tumoral competition
Source: Commun Med (Lond). 2022 Apr 25;2:46. doi: 10.1038/s43856-022-00110-x (PMC9053239; doi:10.1038/s43856-022-00110-x)
Supplement: Supplementary file 3 — Description of Additional Supplementary Files [file 43856_2022_110_MOESM3_ESM.pdf]

Description of Additional Supplementary Files

File Name: Supplementary Movie 1

Description: Example simulation showing the spatiotemporal evolution of the same tumour treated once continuously and once adaptively (Parameters as in Figure 2a).

File Name: Supplementary Movie 2

Description: Example simulations illustrating the dynamics under continuous and adaptive therapy for different values of cost and turnover.

File Name: Supplementary Movie 3

Description: Example simulations of the inferred treatment dynamics of a fast cycling patient (Patient 75), and a slow cycling patient (Patient 88), when fitting the 4-parameter model.

File Name: Supplementary Movie 4

Description: Examples of the inferred, spatiotemporal treatment dynamics for different patients across the cost-turnover space. This illustrates how patients with faster cycles are associated with higher cost, lower turnover values, and more diffuse tumour architectures, whereas patients with slower cycles typically have higher turnover, smaller cost, and a more clustered (“carpet-like”) appearance. Fits shown are those of the 2-parameter model in which only cost and turnover are assumed to be patient-specific.
