# Supplementary material for: Spatial structure impacts adaptive therapy by shaping intra-tumoral competition
Source: Commun Med (Lond). 2022 Apr 25;2:46. doi: 10.1038/s43856-022-00110-x (PMC9053239; doi:10.1038/s43856-022-00110-x)
Supplement: Supplementary file 8 — Reporting Summary [file 43856_2022_110_MOESM8_ESM.pdf]

nature portfolio

Corresponding author(s): Alexander A R Anderson

Last updated by author(s): March, 14, 2022

Reporting Summary

Nature Portfolio wishes to improve the reproducibility of the work that we publish. This form provides structure for consistency and transparency in reporting. For further information on Nature Portfolio policies, see our Editorial Policies and the Editorial Policy Checklist.

Statistics

For all statistical analyses, confirm that the following items are present in the figure legend, table legend, main text, or Methods section.

n/a	Confirmed	
☐	☒	The exact sample size (*n*) for each experimental group/condition, given as a discrete number and unit of measurement
☐	☒	A statement on whether measurements were taken from distinct samples or whether the same sample was measured repeatedly
☐	☒	The statistical test(s) used AND whether they are one- or two-sided<br>*Only common tests should be described solely by name; describe more complex techniques in the Methods section.*
☒	☐	A description of all covariates tested
☒	☐	A description of any assumptions or corrections, such as tests of normality and adjustment for multiple comparisons
☐	☒	A full description of the statistical parameters including central tendency (e.g. means) or other basic estimates (e.g. regression coefficient) AND variation (e.g. standard deviation) or associated estimates of uncertainty (e.g. confidence intervals)
☐	☒	For null hypothesis testing, the test statistic (e.g. *F*, *t*, *r*) with confidence intervals, effect sizes, degrees of freedom and *P* value noted<br>*Give P values as exact values whenever suitable.*
☒	☐	For Bayesian analysis, information on the choice of priors and Markov chain Monte Carlo settings
☒	☐	For hierarchical and complex designs, identification of the appropriate level for tests and full reporting of outcomes
☒	☐	Estimates of effect sizes (e.g. Cohen's *d*, Pearson's *r*), indicating how they were calculated

Our web collection on statistics for biologists contains articles on many of the points above.

Software and code

Policy information about availability of computer code

Data collection	The model is implemented in Java 1.8. using the Hybrid Automata Library. The patient data were downloaded from http://www.nicholasbruchovsky.com/clinicalResearch.html in July 2020. The data underlying all the figures presented in the paper (aside from the patient data which are available from the aforementioned webpage) are available from figshare at: https://doi.org/10.6084/m9.figshare.c.5657311.v4
Data analysis	Data analysis was carried out in Python 3.6, using Pandas 0.23.4, Matplotlib 2.2.3, Seaborn 0.9.0, lmfit 1.0.1., and openCV 3.4.9. The time-evolution of the resistant cells' neighbourhood composition was visualised using EvoFreq in R 4.0.2. The code to carry out all analyses and create all figures presented in the paper is available at https://github.com/MathOnco/strobl2021_space_modulates_competition_AT.

For manuscripts utilizing custom algorithms or software that are central to the research but not yet described in published literature, software must be made available to editors and reviewers. We strongly encourage code deposition in a community repository (e.g. GitHub). See the Nature Portfolio guidelines for submitting code & software for further information.

Data

Policy information about availability of data

All manuscripts must include a data availability statement. This statement should provide the following information, where applicable:

- Accession codes, unique identifiers, or web links for publicly available datasets
- A description of any restrictions on data availability
- For clinical datasets or third party data, please ensure that the statement adheres to our policy

No new experimental or clinical data were generated in this study. All source data for the main figures within this study are available on Figshare at https://

doi.org/10.6084/m9.figshare.c.5657311.v4, and the code to generate each figure has been deposited on Zenodo at https://doi.org/10.5281/zenodo.5504425. The patient data were downloaded from the author's webpage at http://www.nicholasbruchovsky.com/clinicalResearch.html in July 2020.

Field-specific reporting

Please select the one below that is the best fit for your research. If you are not sure, read the appropriate sections before making your selection.

☒ Life sciences ☐ Behavioural & social sciences ☐ Ecological, evolutionary & environmental sciences

For a reference copy of the document with all sections, see nature.com/documents/nr-reporting-summary-flat.pdf

Life sciences study design

All studies must disclose on these points even when the disclosure is negative.

Sample size

Time-series plots show results from 250 independent simulations per conditions. All other analysis are based on 1000 independent replicates.

Data exclusions

Progression was determined as a 20\% increase from the pre-treatment baseline. However, occasionally it could happen that during adaptive therapy the tumour burden briefly exceeded this target at the end of the first or second off-cycle, due to rapid regrowth of the sensitive cells. As the tumour in these cases was immediately brought back under control upon treatment re-administration, and the focus of our study was progression driven by drug-resistant cells, we neglected these events and only considered a tumour to have progressed if at least 150 days had passed. This criterion was applied to both adaptive and continuous therapy.
Secondly, in the analysis of the patient data, 2 patients were excluded because their PSA dynamics was inconsistent with the reported treatment protocol (Patients 2 & 104). For 8 patients, Model 1 failed to recapitulate the cycling nature of the patients' trajectories, yielding simple straight lines instead (e.g. Patient 52 in Figure S2). Due to this discrepancy it was unclear that the associated parameter values would be representative of the tumour biology, and so we excluded these patients when computing the mean of the parameters taken forward to Models 2 and 3. For the same reasons, we excluded these 8 patients and one additional patient (Patient 13) in the cycling speed analysis of Model 3 shown in Figure 7, which was thus based on the data from 56 patients in total. Excluded patients are marked by a grey background in Figures S2 & S3, respectively.

Replication

We carried out a convergence and consistency analysis to ensure numerical accuracy of our algorithm and to ensure our sample sizes large enough that our results were representative of the stochastic process.

Randomization

As our work includes no experimental component, randomization was not relevant to our study.

Blinding

As our work includes no experimental component, blinding was not relevant to our study.

Reporting for specific materials, systems and methods

We require information from authors about some types of materials, experimental systems and methods used in many studies. Here, indicate whether each material, system or method listed is relevant to your study. If you are not sure if a list item applies to your research, read the appropriate section before selecting a response.

Materials & experimental systems

n/a	Involved in the study	
☒	☐	Antibodies
☒	☐	Eukaryotic cell lines
☒	☐	Palaeontology and archaeology
☒	☐	Animals and other organisms
☒	☐	Human research participants
☒	☐	Clinical data
☒	☐	Dual use research of concern

Methods

n/a	Involved in the study	
☒	☐	ChIP-seq
☒	☐	Flow cytometry
☒	☐	MRI-based neuroimaging
